# Facial Diplegia in Bell's Palsy: A Diagnostic Conundrum

**DOI:** 10.7759/cureus.86070

**Published:** 2025-06-15

**Authors:** Yosof Katiby, Jimmy Mcdermott, Lisa Sovory

**Affiliations:** 1 Neurology, Arrowhead Regional Medical Center, Colton, USA; 2 Neurology, California University of Science and Medicine, Colton, USA

**Keywords:** bell’s palsy, cranial nerve vii palsy, facial diplegia, facial nerve paralysis, idiopathic bilateral simultaneous facial nerve palsy

## Abstract

Bell's palsy typically causes one-sided facial paralysis, while bilateral involvement (facial diplegia) is uncommon. The following case is presented of a middle-aged woman who developed simultaneous facial diplegia diagnosed as Bell's palsy. Further complicating the diagnostic picture were MRI findings of temporal lobe abnormalities, recent Botox injection, and unspecified finger nodules. Diagnostic tests excluded other causes of bilateral facial paralysis. The patient was treated with antivirals and steroids and had a full recovery. This case shows the rare occurrence of facial diplegia in Bell's palsy, emphasizing the importance of considering this diagnosis, even in atypical presentations.

## Introduction

Bell's palsy often causes acute peripheral facial nerve palsy, characterized by unilateral facial weakness. However, bilateral facial palsy (facial diplegia) is rare, accounting for approximately 0.3%-2% of all facial paralysis cases [1]. This atypical presentation can be a diagnostic challenge, as it requires excluding various systemic and neurological conditions that can manifest with bilateral facial weakness. The etiological consideration is vast and includes causes such as Bickerstaff brainstem encephalitis, Guillain-Barré syndrome, Miller Fisher syndrome, sarcoidosis, and Lyme disease. A case of a middle-aged woman diagnosed with Bell's palsy who presented with bilateral simultaneous facial weakness is presented, emphasizing the importance of a comprehensive diagnostic approach and consideration of less common causes.

## Case presentation

A 56-year-old female with a history of ovarian cyst and colonic polyps presented to the Emergency Department for a 12-hour history of left-sided facial droop and bilateral facial tingling, which occurred following a stressful conversation the night before. The patient also reported utilizing Botox treatment; however, per family at the bedside, this is not her baseline. Her last utilization of Botox was two months prior, and she describes sporadic usage prior to admission. The patient reports that the paralysis was initially forehead-sparing, but later involved an eyebrow. The patient reported a one-year history of intermittent mild cognitive changes, including word-finding difficulty with substitutions, and musculoskeletal symptoms, including erythema, joint swelling, cutaneous nodules on the distal interphalangeal (DIP) joints of her hands, weakness, and abdominal pain. She had undergone numerous diagnostic studies, including pelvic magnetic resonance imaging, transvaginal ultrasound, thyroid ultrasound, upper and lower endoscopy, and mammography. Notably, she had not done a rheumatological workup. The extensive diagnostic testing resulted in finding a left thyroid nodule, colonic polyps, fatty liver, and adnexal cyst, which have remained stable for many years prior to this admission.

Physical exam findings

**Figure 1:**
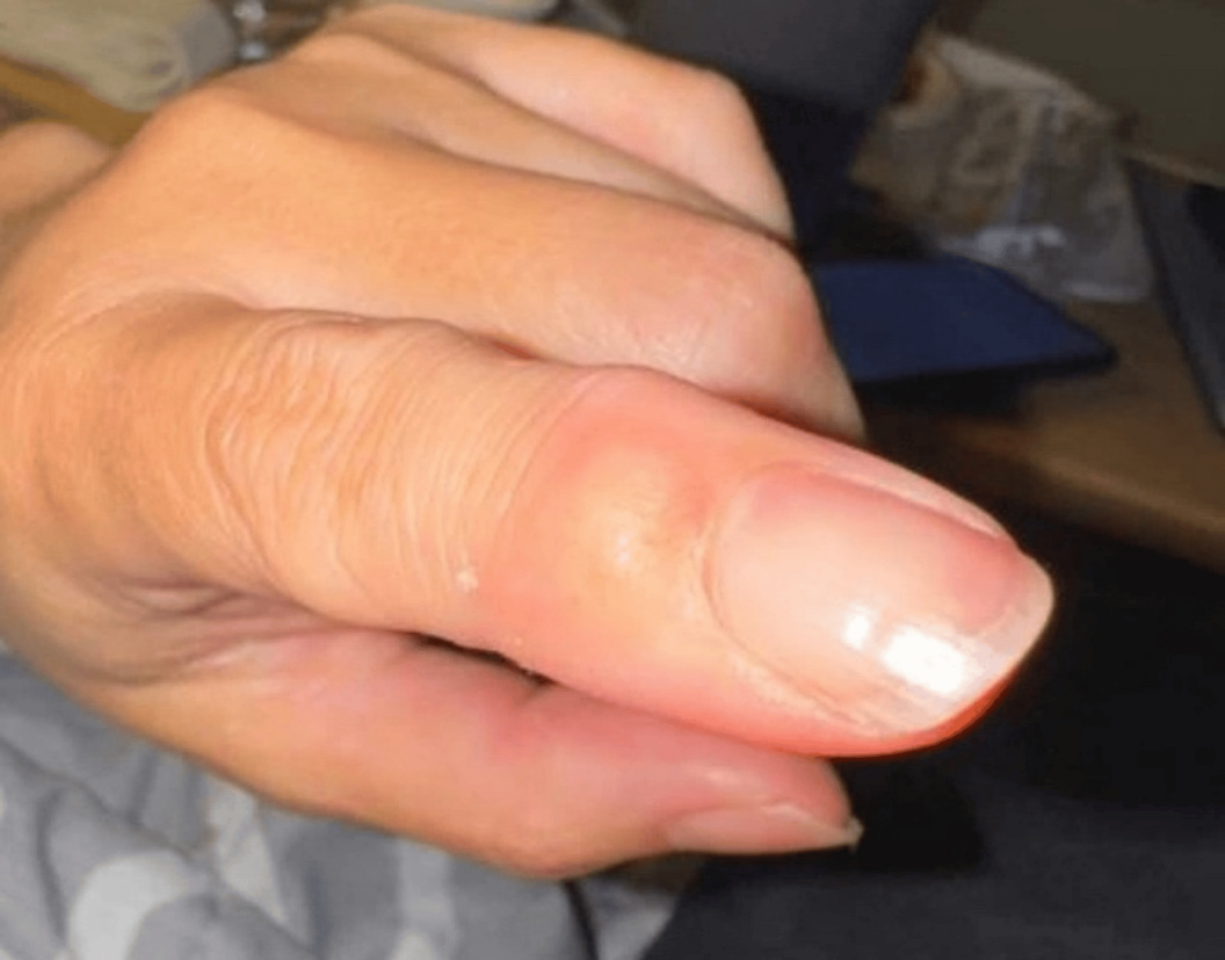
1 cm nodule of the left distal interphalangeal (DIP) joint

The patient was mildly hypertensive on admission, 156/74, but otherwise hemodynamically stable on room air. Neurologic examination revealed some mild speech interruptions with pressured speech and facial diplegia of the upper and lower face. The left face was more affected than the right, with a subtle rightward tongue deviation and left facial nerve V2 branch paresthesia. Reflexes were 3+ bilaterally with bilaterally crossed adductors. Lid lag was present. Musculoskeletal examination revealed a 1 cm immobile nodule overlying the left DIP joint of her second phalanx (Figure 1). Inspection of the ear canal did not show any lesions, pus, or erythema with visualization of the cone of light. The remaining portions of the physical examination were unremarkable.

Evaluation

**Figure 2:**
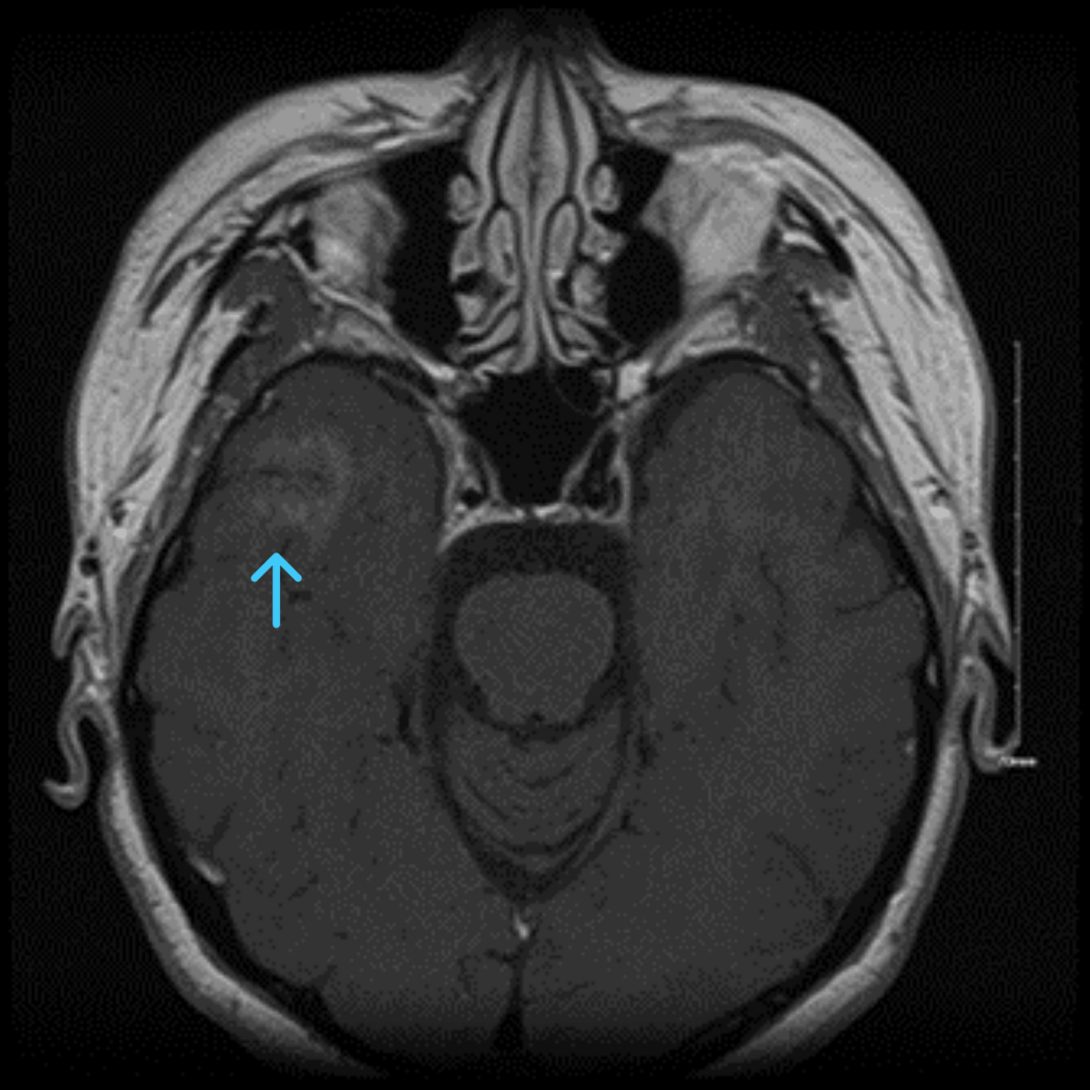
Focal right temporal lobe enhancement on post contrast axial magnetic resonance imaging

**Figure 3:**
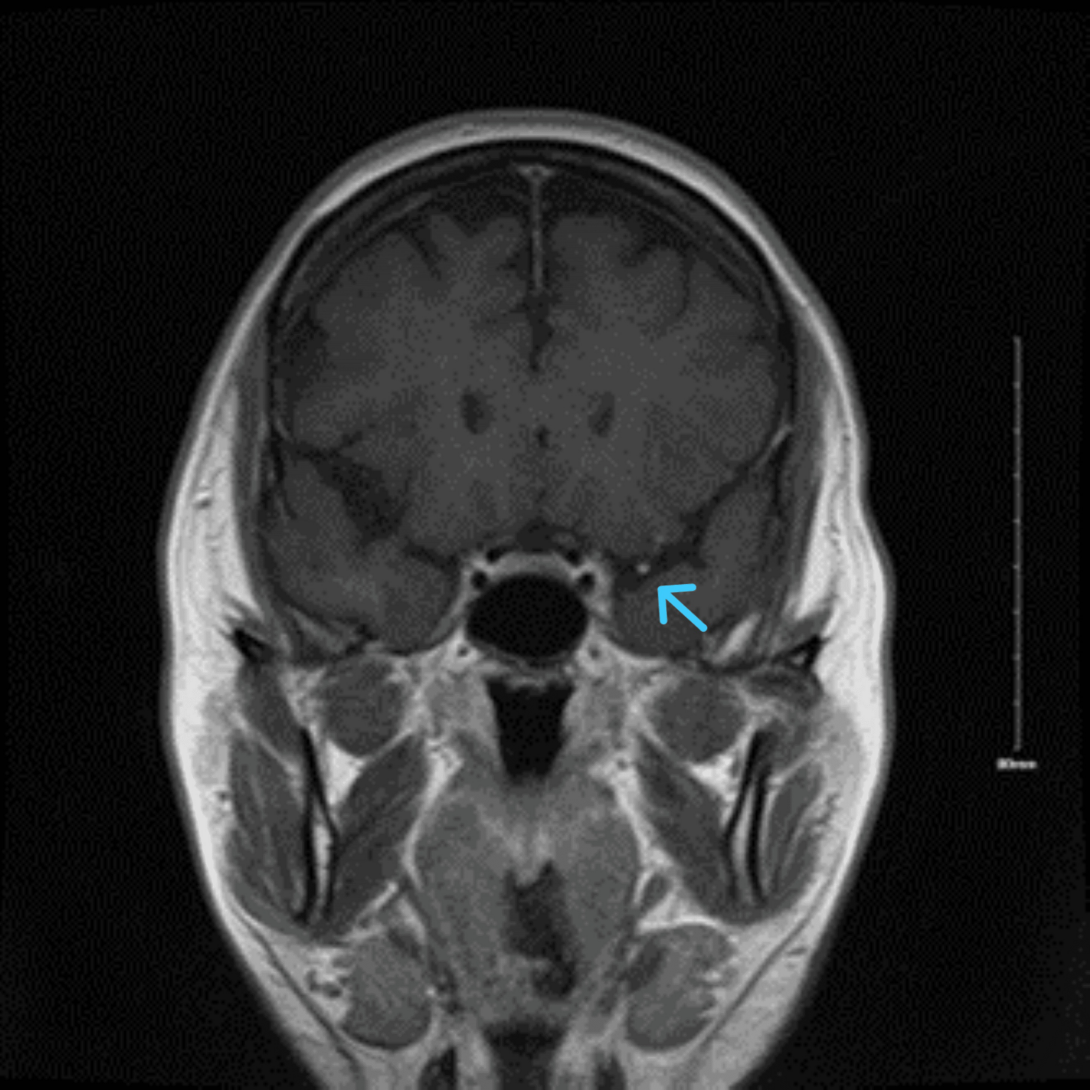
Left facial nerve enhancement on post contrast coronal magnetic resonance imaging

MRI brain internal auditory canal (IAC) protocol with and without contrast revealed focal enhancement of the right temporal lobe with enhancement of the left facial nerve near the level of the internal auditory canal (Figures 2-3). Given a prior history of cyst polyps with focal enhancement, a metastatic workup was pursued, including CT chest and abdomen pelvis with contrast, which did not reveal new abnormalities from medical history. An EEG was also obtained, which did not show any epileptiform activity. Further workup was pursued, which revealed a positive antinuclear antibody screening with a titer of 1:40 and an antinuclear antibody titer of 1:80 but otherwise unremarkable. Lyme PCR and IgG were negative. A lumbar puncture study revealed no evidence of oligoclonal bands, bacterial, viral, and fungal etiology. In addition, cytology was negative for malignant cells. Serum protein electrophoresis did not show any abnormality. Tuberculosis and angiotensin-converting enzyme (ACE) profiles were negative. Initial laboratory findings were unremarkable, including complete blood count (CBC), comprehensive metabolic panel (CMP), thyroid-stimulating hormone (TSH), hepatitis panel, HgbA1C, and urinalysis (Table 1). A biopsy of the skin nodule did not show evidence of granulomas or inflammation (Table 2). Given MRI findings of focal enhancement of the right temporal lobe, an encephalitis workup was pursued, which was unremarkable (Table 3).

**Table 1:** Lab work BUN: blood urea nitrogen, CBC: complete blood count, CMP: comprehensive metabolic panel, MCV: mean corpuscular volume, TSH: thyroid-stimulating hormone

Test	Latest Ref Rng	Result	Flag	
WBC	4.5-11.2 10^3^/uL	5.9		
RBC	4.00-5.20 10^6^/uL	4.30		
Hemoglobin	11.5-15.5 g/dL	13.7		
Hematocrit	36-46%	40		
MCV	80.0-100.0 fL	94		
MCH	26.0-32.0 pg	31.9		
Sodium	135-148 mmol/L	138		
Potassium	3.5-5.5 mmol/L	4.6		
Chloride	98 - Broker 110 mmol/L	104		
CO_2_	24-34 mmol/L	22	Low	
BUN	8-20 mg/dL	11		
Creatinine	0.50-1.50 mg/dL	0.55		
Color	Urine	Dark yellow, Green, Other, Straw, Yellow	Yellow	
Clarity	Urine	Clear	Clear	
pH	Urine	5.0 - 8.0	7.0	
Leukocytes Esterase	Negative	Trace		
Hepatitis B Surface Ag	Non-reactive	Non-reactive		
Hep A IgM	Non-reactive	Non-reactive		
Hep B Core IgM	Non-reactive	Non-reactive		
Hepatitis C Ab	Non-reactive	Non-reactive		
HgbA1C		5.50		
TSH		0.85		

**Table 2:** Workup results ACE: angiotensin-converting enzyme, ANA: anti-nuclear antibody, ANCA: anti-neutrophil cytoplasmic antibody, HSV: herpes simplex virus

Test	Result	Value
ANA Screening	Positive	1:40
Antinuclear Ab Titer	Positive	1:80
ANCA	Negative	
Anti-Smith	Negative	
Sjogrens	Negative	
Scleroderma SM/RNP	Negative	
Crithidia Ab	Negative	
Centromere Ab	Negative	
Cyclic Citrullinated Peptide	Negative	
C3 Complement	Negative	
C4 Complement	Negative	
Lumbar Puncture - Oligoclonal Bands	Negative	
Lumbar Puncture - Bacterial Etiology	Negative	
Lumbar Puncture - Viral Etiology	Negative	
Lumbar Puncture - Fungal Etiology	Negative	
Lumbar Puncture - HSV Studies	Negative	
Lumbar Puncture - Cytology	Negative	
Serum Protein Electrophoresis	Normal	
TB Profile	Negative	
ACE Profile	Negative	
Skin Nodule Biopsy	Negative for Granulomas	
Skin Nodule Biopsy	Negative for Inflammation	

**Table 3:** Encephalitis workup CBA: cell binding assay, DPPX: dipeptidyl-peptidase-like protein 6, CASPR2 Ab: contactin-associated protein-like 2 antibody, LGI1: leucine-rich, glioma-inactivated 1, GABABR: gamma-aminobutyric acid B receptor, NMDAR1 Ab: N-methyl-D-aspartate receptor antibody

Test	Result
Histone Antibodies	Negative
NMDAR1 Ab, CBA	Negative
AMPAR1 Ab, CBA	Negative
AMPAR2 Ab, CBA	Negative
GABABR Ab, CBA	Negative
LGI1 Ab, CBA	Negative
CASPR2 Ab, CBA	Negative
DPPX Receptor Ab, CBA IFA	Negative

Hospital course

On the day of admission, the patient was started on acyclovir IV every eight hours and solumedrol daily. Acyclovir treatment was discontinued after negative CSF studies. The patient reported subjective improvement of speech and facial paralysis but was still objectively noted. The patient continued on IV Solu-Medrol for five days. On the last day of treatment, facial involvement was mainly localized to the left face, with resolution of the right face. Repeat MRI brain with and without contrast demonstrated similar findings to the initial MRI. The patient was discharged to outpatient follow-up. When seen in an outpatient setting three months later, complete resolution was noted; however, she reported resolution four weeks after discharge, and no repeat MRI brain was obtained as symptoms had resolved.

## Discussion

Bell's palsy, typically a unilateral condition, is a common cause of facial nerve weakness, often attributed to inflammation and swelling of the facial nerve. While bilateral Bell's palsy can occur, it is considerably less frequent than its unilateral counterpart and may suggest an underlying systemic issue. Facial diplegia is a rare condition with an incidence of one in 5,000,000 cases [2]. This case presents an uncommon finding of imaging abnormalities combined with a presentation of facial diplegia and unremarkable workup. The absence of typical Bell's palsy symptoms (e.g., facial pain, altered taste) in this case, coupled with the bilateral presentation and temporal lobe abnormality, warrants a thorough investigation for alternative secondary causes. We highlight the potential for further investigation into bilateral seventh nerve palsy.

Differential diagnoses

The differential diagnosis for bilateral facial diplegia is broad and includes infectious, inflammatory, neurological, neoplastic, and traumatic causes. The most common of these causes are Guillain-Barré syndrome, Lyme disease, sarcoidosis, and tumor compression. Rarer considerations would be encephalitis and paraneoplastic causes. While Bell's palsy is a consideration, it is a diagnosis of exclusion after all other etiologies are exhausted.

Guillain-Barré syndrome is a significant consideration, especially in cases with rapidly progressive or symmetrical weakness and areflexia. It typically presents with other systemic neurological findings, such as absent reflexes and decreased strength/sensation on examination. Case reports have been found to have isolated facial diplegia, although in those cases, reflexes were found to be minimal or absent. Only after the facial nerve conduction study was a diagnostic confirmation achieved [3]. Given the response to steroids and antivirals and lack of progression of the disease, this makes Guillain-Barré syndrome unlikely, as typically IVIG or plasmapheresis would improve Guillain-Barré syndrome.

Lyme disease, a tick-borne illness, is another important differential, particularly in endemic areas, and can manifest with bilateral facial paralysis. Case reports of Lyme disease presenting with facial diplegia exist, although accompanied by the typical erythema migrans physical examination finding. Diagnosis of Lyme disease with facial diplegia necessitates doxycycline treatment [4]. Other potential causes include sarcoidosis, a systemic inflammatory disease that can affect the nervous system, and, less frequently, tumors affecting the facial nerves or brainstem.

Rare autoimmune encephalitis have been shown to cause facial diplegia, such as Miller Fisher, brainstem encephalitis, and carcinomatous meningitis. Given the MRI abnormalities in our case, the diagnosis of encephalitis was strongly considered. A case series of 62 cases of Bickerstaff brainstem encephalitis highlights the presence of facial diplegia in approximately 42% of cases [5]. Although brainstem encephalitis typically presents with other symptomatologies of ataxia, ophthalmoplegia, and altered mentation. In addition, paraneoplastic causes can be a consideration, although limited case reports exist. Paraneoplastic causes typically involve breast cancer or small cell lung cancer and are anti-amphiphysin positive. Even then, the patients are typically already diagnosed with malignancy rather than a herald of a coming malignancy. Treatment is typically with steroids and IVIG, with partial response to treatment noted [6]. Given the MRI abnormalities, encephalitis would be a consideration in this case; however, the encephalitis panel, as highlighted in the case presentation, was negative. As far as we are aware, no paraneoplastic cause of facial diplegia heralding a malignancy has been identified.

It is questionable whether the temporal lobe anomaly found in our patient was a cause of the facial nerve palsy; however, previous reports of facial nerve palsy secondary to temporal lobe involvement exist. In one case, after temporal lobectomy, it was theorized that the inflammation and stress of the procedure could cause a reactivation of latent herpesvirus within the geniculate ganglion, leading to involvement of the facial nerve. Other case reports reveal compressive etiologies, such as malignant peripheral nerve sheath tumor, leading to persistent facial nerve palsy in the temporal bone region in patients with recurrent facial nerve palsy or persistent worsening of symptoms [7]. In this case, investigations for these conditions were either negative or non-contributory.

Given the extensive workup and lack of definitive findings, it is possible that this case represents a rare, idiopathic form of bilateral facial paralysis or autoimmune etiology yet to be identified by antibody testing. The absence of preceding infection or specific risk factors makes pinpointing a precise etiology challenging. In addition, the MRI abnormality further complicates the clinical picture. However, the patient's clinical course and response to treatment provide valuable insights. The spontaneous and gradual improvement suggests a potential inflammatory or autoimmune process affecting the facial nerves, even in the absence of specific serological markers.

Management and prognosis

The patient received corticosteroids and antivirals, a commonly used treatment for Bell's palsy and other inflammatory conditions affecting the facial nerves. The favorable response to corticosteroids supports the hypothesis of an inflammatory etiology, although this remains speculative. Long-term management includes facial physiotherapy to prevent muscle contractures and support functional recovery [8].

## Conclusions

While most facial nerve palsies are idiopathic, it is important to note that Bell’s palsy is a diagnosis of exclusion and other causes of cranial nerve involvement must be ruled out before a definitive diagnosis can be made. Facial diplegia itself is an ominous presentation of facial nerve palsy as it implies an underlying systemic illness. While rarely presented bilaterally, a thorough evaluation, history, physical, and diagnostic workup should be pursued in patients with this rare variant, as the likelihood of a missed underlying cause is especially greater. This case demonstrated a rare and complicated presentation of Bell's palsy with facial diplegia.
